# Editorial: Community-Based Outreach Treatment for Addictions and Concomitant Disorders: Time for a Change of Paradigm

**DOI:** 10.3389/fpsyt.2020.00002

**Published:** 2020-01-22

**Authors:** Louise Penzenstadler, Yasser Khazaal, Marie-Josée Fleury

**Affiliations:** ^1^ Department of Psychiatry, Geneva University Hospitals, Geneva, Switzerland; ^2^ Addiction Medicine, Department of Psychiatry, Lausanne University Hospitals, Lausanne, Switzerland; ^3^ Research Center, Montreal University Institute of Mental Health, Montreal, QC, Canada; ^4^ Department of Psychiatry, Douglas Mental Health University Institute (Research Center), McGill University, Montreal, QC, Canada

**Keywords:** addiction, concomitant disorders, community-based treatment models, recovery, paradigm change

In addiction medicine, traditional outreach treatment mainly aims at risk reduction, for example, of overdose and infectious diseases. Besides, and complementary to, risk reduction treatments, other needs could be more adequately addressed, such as social rehabilitation and inclusion (1, 2). Over the last few decades, different models of community treatment for individuals with substance use disorders (SUD) and concomitant disorders have been developed (3, 4). Target populations often present complex disorders and face difficult social situations and frequently present high emergency and inpatient service use (5). This suggests that most outpatient programs are often unsuccessful at addressing the needs of this population.

Recently, newer models of community-based outreach treatment have been developed in psychiatry using recovery-oriented practices (6), in accordance with the United Nations Convention on the Rights of Persons with Disabilities (7). Central to recovery-oriented service delivery is the idea that, as well as offering evidence-based treatments, services have to adapt to people's needs, instead of people to service requirements (8). Among others, “housing first” and “supported employment” have been regarded as successful examples of the implementation of such a recovery model in mental health services (9, 10). Only few studies have examined specific community-based outreach treatment models for patients with addictions and concomitant disorders, especially with respect to recovery-oriented practices. Most of these studies either excluded or did not specifically assess the effect of these treatment models for individuals with SUD.

In light of these considerations, new models of addiction treatment are generally moving from prevention and risk reduction-based models to community treatment models which focus on inclusion and recovery (11). In order to further consolidate this paradigm change, the factors associated with the recovery process and with the stakeholders' possible views and barriers need to be studied as well. Therefore, the aim of this Research Topic is to present and discuss some examples of recovery-oriented models for individuals with addictions and concomitant disorders. The Research Topic includes eight articles which study new treatment models and service organization from different angles. They discuss service organization, different treatment models, and their possibilities for improvement, as well as how to facilitate access to care with the use of new technologies.

Existing community treatment programs, such as case management for individuals with SUD, are reviewed in the meta-analysis by Vanderplasschen et al. from a recovery-oriented perspective: previous findings of improved treatment retention for patients receiving case management are confirmed. However, further research is needed to assess the recovery potential of case management. The review by Wiktorowicz et al. of policies, service coordination, and access issues illustrates the remaining differences and challenges in the implementation of collaborative care models and local networks to foster service coordination for concurrent disorders. In order to understand which factors may be improved by community-based treatment, Gentil et al. investigated different profiles of homeless populations using clusters analysis. They found that SUD, mental health disorders, and high functional disability (complex disorders) had an important impact on quality of life, a fact which needs to be considered when designing community treatment programs for marginalized populations.

The study by Yang et al. has shown that, while programs need to be adapted to different populations, it is also important to improve the perceived social support and resilience in order to improve life satisfaction among individuals with SUD. When further exploring factors associated with recovery in community interventions, stakeholders' attitudes, and views also have to be examined. The detailed analysis of recovery attitudes and practices in an assertive community treatment team (ACT) by Khoury helps to understand which practices are associated with recovery. Especially the interactions based on collaborative egalitarian relationships between service providers and service users seem to be especially promising in the innovation of practice approaches in this field.

Another important goal of community-based outreach programs is to enable access for hard-to-reach populations and individuals who do not normally use addiction or mental health services. Such programs are reviewed by Edalati and Conrod, who examined modifiable risk factors which may lead to SUD and personality-targeted community interventions for adolescents aimed at reducing substance misuse. These interventions were found to facilitate access to care at school, as high-risk adolescents may not have the possibility to access other services. They contributed to reduce substance misuse and concomitant mental health problems. The study by Flores-Aranda et al. also describes an intervention for individuals not usually reached by traditional SUD programs. They presented an online intervention for men who have sex with men which aims to prevent substance use and sexual health related problems. Online tools have gained popularity in various fields of health prevention and treatment and could be helpful in facilitating access to care Monney et al. The literature review by Pennou et al. further highlights the treatment possibilities *via* mobile phone of core difficulties such as emotional regulation found in individuals with dual disorders.

The diversity of these contributions shows important promises in community-based outreach treatment for addictions. The topic highlights a major paradigm change in addiction treatment, moving away from a stepwise approach of harm reduction and relapse prevention followed by work on social inclusion. The new paradigm focuses on inclusion and recovery from the beginning and implements a more integrated approach with a range of interventions and goals which meet diverse needs aimed at enabling a sustained recovery management. Further research is needed on how to best translate recovery-oriented practices from theoretical aims to service organization and clinical practices and how to best include service users in this process.

## Author Contributions

LP wrote the first draft of the manuscript. YK and M-JF provided critical revision of the manuscript and important intellectual contributions. All authors read and approved the submitted version.

## Conflict of Interest

The authors declare that the research was conducted in the absence of any commercial or financial relationships that could be construed as a potential conflict of interest.

## References

[1] Heilig M Epstein DH Nader MA Shaham Y Time to connect: bringing social context into addiction neuroscience. Nat Rev Neurosci (2016) 17:592–9. 10.1038/nrn.2016.67 PMC5523661 27277868

[2] Kiluk BD Fitzmaurice GM Strain EC Weiss RD What defines a clinically meaningful outcome in the treatment of substance use disorders: reductions in direct consequences of drug use or improvement in overall functioning? Addiction (2019) 114:9–15. 10.1111/add.14289 29900624 PMC6289694

[3] Penzenstadler L Soares C Anci E Molodynski A Khazaal Y Effect of assertive community treatment for patients with substance use disorder: a systematic review. Eur Addict Res (2019) 25:56–67. 10.1159/000496742 30699412

[4] Rapp RC Van Den Noortgate W Broekaert E Vanderplasschen W The efficacy of case management with persons who have substance abuse problems: a three-level meta-analysis of outcomes. J Consulting Clin Psychol (2014) 82:605–18. 10.1037/a0036750 24821097

[5] Fleury M-J Rochette L Grenier G Huỳnh C Vasiliadis H-M Pelletier É Factors associated with emergency department use for mental health reasons among low, moderate and high users. Gen Hosp Psychiatry (2019) 60:111–9. 10.1016/j.genhosppsych.2019.07.006 31404825

[6] Slade M Amering M Farkas M Hamilton B O'Hagan M Panther G Uses and abuses of recovery: implementing recovery-oriented practices in mental health systems. World Psychiatry (2014) 13:12–20. 10.1002/wps.20084 24497237 PMC3918008

[7] United Nations Convention on the Rights of persons with disabilities. (New York: United Nations). (2006).

[8] Frost BG Tirupati S Johnston S Turrell M Lewin TJ Sly KA An Integrated Recovery-oriented Model (IRM) for mental health services: evolution and challenges. BMC Psychiatry (2017) 17:22. 10.1186/s12888-016-1164-3 28095811 PMC5240195

[9] Urbanoski K Veldhuizen S Krausz M Schutz C Somers JM Kirst M Effects of comorbid substance use disorders on outcomes in a Housing First intervention for homeless people with mental illness: effectiveness of housing first. Addiction (2018) 113:137–45. 10.1111/add.13928 28667822

[10] Aklin WM Wong CJ Hampton J Svikis DS Stitzer ML Bigelow GE A therapeutic workplace for the long-term treatment of drug addiction and unemployment: eight-year outcomes of a social business intervention. J Subst Abuse Treat (2014) 47:329–38. 10.1016/j.jsat.2014.06.013 PMC4176507 25124257

[11] El-Guebaly N The meanings of recovery from addiction: evolution and promises. J Addict Med (2012) 6:1–9. 10.1097/ADM.0b013e31823ae540 22124289

